# Enzymatically treated yeast bolstered growth performance of broiler chicks from young broiler breeders linked to improved indices of intestinal function, integrity, and immunity

**DOI:** 10.1016/j.psj.2022.102175

**Published:** 2022-09-13

**Authors:** Anderson N. Maina, Aizwarya Thanabalan, Jessica Gasarabwe, Mohsen Mohammadigheisar, Hagen Schulze, Elijah G. Kiarie

**Affiliations:** ⁎ Department of Animal Biosciences, University of Guelph, Guelph, ON N1G 2W1, Canada; # Livalta, AB Agri Ltd., Peterborough, UK

**Keywords:** broiler breeder age, broiler chickens, growth performance, enzymatically treated yeast, gut health

## Abstract

Older breeder chicks (**OBC**) are heavier and robust at hatch than younger breeder chicks (**YBC**). However, the implications of broiler breeder age on chick intestinal function and the role of functional feedstuffs are unexplored. We evaluated the effects of broiler breeder age and the impact of feeding YBC enzymatically treated yeast on growth, nutrient utilization, and indices of intestinal function. Fertile Ross 708 eggs: 2,250 (56.5 ± 3.4g) from 30-wk-old (YBC) and 550 (64.2 ± 4.2 g) from 47-wk-old (**OBC**) were hatched and placed in 48 pens (44 chicks/pen) containing equal males and females for growth and intestinal function evaluation and 36 cages (5 chicks/cage) for metabolizable energy (**AME**). Five corn and soybean meal-based diets were formulated to contain 0, 0.05, 0.10, 0.20, and 0.40% HY40 for a 3-phase feeding program (starter: days 0–10, grower: days 11–24, and finisher; days 25–42). Grower phase diets also contained a 0.3% TiO_2_ indigestible marker. The diets were allocated within YBC in a completely randomized block design (n = 8 for pens; n = 6 for cages). The OBC were fed a 0% yeast diet. Feed and water were provided freely; BW and feed intake were monitored, and excreta samples were collected on days 17 to 21 for apparent retention (**AR**). Birds were necropsied for plasma, jejunal tissues, organs weight, and ceca digesta. The OBC were heavier (*P* < 0.01) than YBC at hatch. Final BW of OBC and YBC fed, ≥0.10% yeast, was similar (*P* > 0.05). The OBC had similar FCR (*P* > 0.05) to YBC fed 0 to 0.10% yeast but higher (*P* = 0.003) than for YBC fed ≥0.20% yeast. Jejunal villi height to crypt depth ratio (**VCR**) and IgA were higher in OBC than 0% yeast (*P* = 0.01). Yeast increased VCR, bursa weight, jejunal, and plasma IgA (*P* = 0.01). The YBC fed ≥0.10% yeast had higher (*P* < 0.05) AR of crude protein, and gross energy than OBC and YBC fed 0 or 0.05% yeast. In conclusion, yeast improved YBC performance to the level of OBC linked to improved intestinal function, integrity, and immunity.

## Introduction

Broiler breeder age is a crucial factor affecting progeny performance. Older breeders lay heavier eggs than younger breeders due to their much-developed reproductive and physiological maturity. Studies have shown that larger eggs hatch heavier chicks that shows superior post-hatch growth performance within at least ten days of live (Ulmer-Franco et al., 2010; Duman and Şekeroğlu, 2017). Whereas several studies (Sklan et al., 2003; Ulmer-Franco et al., 2010) have shown that chick weight is an essential factor in the performance of broiler chicks to market weight, Wilson (1991) reported that any advantage of chicks hatched from large-sized eggs diminishes rapidly after hatching. In addition, the mortality of broiler chickens hatched from different sizes of eggs is also variable (Iqbal et al., 2017). Broiler chick characteristics at hatch are therefore affected by breeder physiology, including but not limited to breeder age, thereby influencing chick weight, growth, intestinal development, and immunocompetence (Mahmoud and Edens, 2012; Ipek and Sozcu, 2014). Generally, lighter chicks experience adverse effects such as metabolic disorders, depressed effectiveness of the overall immune system, and decreased resistance to pathogenic loads.

Yeast metabolites have been reported to enhance growth performance indices, immunocompetence, and resilience against enteric pathogens of economic importance in poultry production, such as *Eimeria* and *Clostridium perfringens* (Kiarie et al., 2019, 2022). The growth-enhancing and immune-modulatory potential of yeast products in poultry production has been reported (Kareem et al., 2016; Jacob and Pescatore, 2017). Of particular interest in yeast are the functional components of cell contents such as peptides, enzymes, nucleotides, and cell wall constituents such as β-glucans, glycoproteins, α-mannans, and chitin (Cabib et al., 2008). However, there is a dearth of data demonstrating the benefits of feeding enzymatically treated yeast to close the growth and health performance gap between chicks from broiler breeders differing in age. Therefore, the objectives of this study were to investigate the effects of feeding enzymatically treated yeast (HY40) on growth performance, intestinal function, and gut ecology in broiler chicks (**YBC**) from young broiler breeders. In addition, the chicks from older broiler breeders (**OBC**) were included in the study as a positive control and to record comparative differences in intestinal ecology and function with YBC.

## MATERIALS AND METHODS

The study was carried out according to the Canadian Council on Animal Care guidelines (CCAC, 2009), and animal care and user protocols were approved by the University of Guelph Animal Care Committee (protocol # 4403).

### Birds, Housing, and Experimental Diets

#### Hatchery

Two batches of fertilized broiler (Ross x Ross 708) eggs were procured from two independent Ross 708 broiler breeder flocks in a commercial farm (Maple Leaf Foods, New Hamburg, ON, Canada). One batch (2,250 eggs) was from a 30-wk-old broiler breeder flock (YBC), and the other batch (550 eggs) was from 47 weeks broiler breeder flock (OBC) from the same broiler breeder farm. The average egg weight was 56.5 ± 3.4 and 64.2 ± 4.2 g for the young and old flock, respectively. The experimental eggs were incubated at 37.5°C with 55 % humidity to day 19 and then transferred to a hatcher set at 36.9°C with 66% humidity (Akbari Moghaddam Kakhki et al., 2020). Upon hatching, the chicks were vaccinated against Mareks disease, coccidiosis, and infectious bronchitis as per Arkell Poultry Research Station standard operating procedures.

#### Floor Pen Trial

Based on hatch BW, 2,112 sexed chicks were placed in pens (44 chicks per pen; equal males and females/pen) housed in four separate environmentally controlled rooms with 12 pens each. Each pen provided a 46 sq. ft area and was bedded with fresh wood shavings. Of the 48 pens, 40 were allocated to YBC and 8 to OBC, randomly distributed throughout the four rooms. The room temperature was set to the breeder recommendation of 32°C on day 0 and gradually decreased to 27°C by day 17 (Aviagen, 2014). Birds were exposed to fluorescent lighting in 23 h of light (20+ lux) for the first four days and then a 16 light: 8 dark (10–15 lux) light cycle for the remainder of the experiment following Arkell Poultry Research Station protocols.

#### Metabolism Cage Trial

The remaining 180 chicks were placed in 36 cages (5 chicks per cage), 6 cages for OBC, and 30 cages for YBC. Allocation for the metabolism trial was after selecting birds for the growth trial. In this context, because of few birds to select from, the sex was not considered in metabolism trial. Each cage (76 cm width, 51 cm depth, and 56 cm height) was equipped with two nipple drinkers connected to a standard water line supplying the whole experimental room and an independent trough feeder (70 cm length, 8.5 cm width, 9 cm depth). The lighting schedule was 23D:1D at 100 lux on day 1, 12L:12D at 30 lux until week 2, and 8L:16D at 30 lux. The experimental room temperature was set at 32°C and gradually decreased to 29°C by day 13.

#### Experimental Diets

The yeast was an enzymatically treated whole non-GMO *Saccharomyces cerevisiae* strain assayed to contain 40% β-1.3/1.6 glucans, mannan oligosaccharides, and 36% crude protein (LivaltaCell HY40, AB AGRI, Peterborough, UK). The diets were formulated for a 3-phase feeding program: starter; days 0 to 10, grower: days 11 to 24, and finisher; days 25 to 42, to meet or exceed the nutrient requirements of Ross x Ross 708 recommended by the breeder (Aviagen, 2014). The diets were: a corn-soybean meal diet (0% yeast) and four other diets created by adding 0.05, 0.10, 0.20, and 0.40% yeast at the expense of corn (Table 1). All diets contained 500 FTU of phytase/kg and were free of anticoccidial, antimicrobial growth-promoting products and alternatives, such as pre- and pro-biotics and phytogenics. The starter diet was prepared in fine crumble form, grower feed was in coarse crumble form, and the finisher was prepared in short pellet form. The temperature of the processing condition was 60 to 65°C with a steam pressure of 30 psi. Samples of feed were collected for nutrient analyses.

**Table 1:** Composition of experimental diets, as fed basis.

Yeast^1^ inclusion, %	Starter (d 0–10)					Grower (d 11–24)					Finisher 1 (d 25–42)				
	0.0	0.05	0.10	0.20	0.40	0	0.05	0.10	0.20	0.40	0	0.05	0.10	0.20	0.40
Corn	48.66	48.61	48.56	48.46	48.26	41.89	41.84	41.79	41.69	41.49	46.45	46.4	46.35	46.25	46.05
Soybean meal	34.7	34.7	34.7	34.7	34.7	28.6	28.6	28.6	28.6	28.6	24.0	24.0	24.0	24.0	24.0
Wheat	7.7	7.7	7.7	7.7	7.7	17.2	17.2	17.2	17.2	17.2	15.0	15.0	15.0	15.0	15.0
Corn gluten meal	0.46	0.46	0.46	0.46	0.46	−	−	−	−	−	−	−	−	−	−
Canola meal	3.00	3.00	3.00	3.00	3.00	5.00	5.00	5.00	5.00	5.00	7.09	7.09	7.09	7.09	7.09
Soybean oil	1.15	1.15	1.15	1.15	1.15	3.08	3.08	3.08	3.08	3.08	3.99	3.99	3.99	3.99	3.99
Limestone	1.40	1.40	1.40	1.40	1.40	1.25	1.25	1.25	1.25	1.25	1.11	1.11	1.11	1.11	1.11
Monocalcium phosphate	0.87	0.87	0.87	0.87	0.87	0.69	0.69	0.69	0.69	0.69	0.52	0.52	0.52	0.52	0.52
Sodium chloride	0.25	0.25	0.25	0.25	0.25	0.26	0.26	0.26	0.26	0.26	0.27	0.27	0.27	0.27	0.27
Sodium bicarbonate	0.17	0.17	0.17	0.17	0.17	0.16	0.16	0.16	0.16	0.16	0.13	0.13	0.13	0.13	0.13
L-Lysine HCl	0.27	0.27	0.27	0.27	0.27	0.25	0.25	0.25	0.25	0.25	0.20	0.20	0.20	0.20	0.20
DL-Methionine	0.30	0.30	0.30	0.30	0.30	0.26	0.26	0.26	0.26	0.26	0.21	0.21	0.21	0.21	0.21
L-Threonine	0.06	0.06	0.06	0.06	0.06	0.05	0.05	0.05	0.05	0.05	0.02	0.02	0.02	0.02	0.02
Vitamin and trace premix^2^	1.00	1.00	1.00	1.00	1.00	1.00	1.00	1.00	1.00	1.00	1.00	1.00	1.00	1.00	1.00
Phytase^3^	0.01	0.01	0.01	0.01	0.01	0.01	0.01	0.01	0.01	0.01	0.01	0.01	0.01	0.01	0.01
Titanium dioxide	−	−	−	−	−	0.30	0.30	0.30	0.30	0.30	−	−	−	−	−
Yeast	0.0	0.05	0.10	0.20	0.40	0.0	0.05	0.10	0.20	0.40	0.0	0.05	0.10	0.20	0.40
Calculated provisions															
AMEn, kcal/kg	3,000	3,000	3,000	3,000	3,000	3,100	3,100	3,100	3,100	3,100	3,200	3,200	3,200	3,200	3,200
Crude protein, %	23.2	23.2	23.2	23.2	23.2	21.3	21.3	21.3	21.3	21.3	20.0	20.0	20.0	20.0	20.0
Crude fat, %	3.72	3.72	3.72	3.72	3.72	5.59	5.59	5.59	5.59	5.59	6.62	6.62	6.62	6.62	6.62
SID Lys, %	1.28	1.28	1.28	1.28	1.28	1.15	1.15	1.15	1.15	1.15	1.03	1.03	1.03	1.03	1.03
SID Met + Cys, %	0.95	0.95	0.95	0.95	0.95	0.87	0.87	0.87	0.87	0.87	0.80	0.80	0.80	0.80	0.80
SID Trp, %	0.26	0.26	0.26	0.26	0.26	0.24	0.24	0.24	0.24	0.24	0.22	0.22	0.22	0.22	0.22
SID Thr, %	0.80	0.80	0.80	0.80	0.80	0.72	0.72	0.72	0.72	0.72	0.65	0.65	0.65	0.65	0.65
Calcium, %	0.96	0.96	0.96	0.96	0.96	0.87	0.87	0.87	0.87	0.87	0.79	0.79	0.79	0.79	0.79
Available phosphorous, %	0.48	0.48	0.48	0.48	0.48	0.44	0.44	0.44	0.44	0.44	0.40	0.40	0.40	0.40	0.40
Sodium, %	0.18	0.18	0.18	0.18	0.18	0.20	0.20	0.20	0.20	0.20	0.18	0.18	0.18	0.18	0.18

1 Enzymatically treated whole non-GMO *Saccharomyces cerevisiae* strain, 40% β-1.3/1.6 glucans and mannan oligosaccharides and 36% crude protein (Livalta TMCell HY40, AB AGRI, Peterborough, UK).

2 Provided per kilogram of diet: vitamin A, 8800.0 IU; vitamin D_3_, 3300.0 IU; vitamin E, 40.0 IU; vitamin B_12_, 12.0 mg; vitamin K_3_, 3.3 mg; niacin, 50.0 mg; choline, 1200.0 mg; folic acid, 1.0 mg; biotin, 0.22 mg; pyridoxine, 3.3 mg; thiamine, 4.0 mg; calcium pantothenic acid, 15.0 mg; riboflavin, 8.0 mg; manganese, 70.0 mg; zinc, 70.0 mg; iron, 60.0 mg; iodine, 1.0 mg; copper, 10 mg; and selenium, 0.3 mg.

3 Provided 500 FTU of phytase per kg feed, providing 0.15% available P and 0.16% Ca (Quantum Blue, AB Vista, Marlborough, UK).

### Experimental Procedures, Measurements, and Sampling

#### Growth Performance (Floor Pens) Trial

Older breeder chicks were fed the control diet (0% yeast), effectively creating 6 treatments. Treatments were allocated in a completely randomized block design (n = 8). Throughout the experiment, experimental birds had free access to water via nipples and fed via feed. Body weight and feed intake were monitored on days 0, 10, 24, and 42 for body weight gain (**BWG**) and feed conversion ratios (**FCR**). In addition, mortalities were counted, and body weights were recorded to measure mortality adjusted FCR.

#### Floor Pen Sample Collection and Processing

On days 10, 24, and 42, two birds (one male and one female) per pen were randomly selected for sampling. For day 10, birds were weighed, bled through cardiac puncture, and euthanized via cervical dislocation. The liver, spleen, and bursa were excised, blotted dry with a paper towel, and weighed. The jejunum was located, and jejunal tissue samples were excised at 10 cm anterior to Meckel's diverticulum for histomorphology and intestinal gene expressions. Histomorphology samples were preserved in buffered formalin for histomorphology, and samples for gene expression were collected in vials filled with one mL of RNAlater, placed on ice, and stored at −80°C until required analyses. For day 24, ceca digesta samples were collected using kits supplied by a commercial laboratory (Alimetric Diagnostics Ltd., Espoo, Finland). The samples were subsequently shipped to Alimetrics for processing and analyses of the concentration of total bacteria and short-chain fatty acids (**SCFA**). For 42, breast weight was recorded from the two birds. Litter samples were collected on days 35 and 42 for litter moisture content. Briefly, samples were collected from the center and mid-way between each pen's center and four corners (Leung et al., 2019).

### Metabolism Cage Trial

The six treatments were randomly assigned to give six replicate cages per treatment. The birds had free access to the diets and water throughout the 21-d experimental period. From day 17 to 21 posthatching, excreta samples were collected per cage to determine the apparent retention of components. Excreta samples were pooled within a cage, resulting in 6 samples per dietary treatment, and frozen immediately after collection until analysis.

### Sample Processing and Analyses

#### Diets and Excreta

Samples for the diets were finely ground and submitted to a commercial lab (S.G.S. Canada, Guelph) for dry matter (**DM**), crude protein (**CP**), crude fat, starch, and minerals analyses. Excreta samples were oven-dried at 60°C to constant weight, and along with grower diet finely ground using a grinder (CBG5 Smart Grind, Applica Consumer Products Inc., Shelton, CT) and thoroughly mixed for chemical analyses. All the samples were analyzed for DM, TiO_2_, gross energy (**GE**), and nitrogen (**N**). Dry matter was determined according to method 930.15 (AOAC, 2005). Nitrogen was determined using combustion method 968.06 (AOAC, 2005) in a CNS-2000 carbon, nitrogen, and sulfur elemental analyzer (Leco Corporation, St. Joseph, MI). The CP was derived by multiplying the assayed nitrogen values by 6.25. Gross energy was determined using bomb calorimeter (IKA Calorimeter System C 5000; IKA Works, Wilmington, NC). The Titanium content was measured on a UV spectrophotometer following Myers et al. (2004). The litter samples were dried for constant weight at 60°C to determine litter moisture.

#### Jejunal Histomorphology

Fixed jejunal tissue samples were cut into a longitudinal cross-section and embedded in paraffin wax. The tissues were then sectioned (5 µm) and stained with hematoxylin and eosin for morphological measurements. Five villous-crypt structures were measured with a calibrated micrometer for each tissue using a Leica DMR microscope (Leica Microsystems, Wetzlay, Germany). Villi height and crypt depth ratio (**VCR**) was calculated.

#### Jejunal Tissue Gene Expression

The total RNA from 50 to 100 mg of jejunal tissues was extracted according to manufacturer instructions (Thermo Fisher Scientific, Mississauga, ON, Canada). The RNA was purified by precipitation with Lithium Chloride and quantified by a Nanodrop ND-2000 spectrophotometer (Thermo Fisher Scientific). The ratio of OD260 and OD280 was between 1.8 and 2.1. The integrity of RNA was verified by visualization in an agarose gel (supplementary Figure 1) and stored at −80°C. To create a cDNA library, 2 μg of total RNA was transcribed into cDNA using the Superscript II kit (BioRad, Hercules, CA), following the manufacturer's instructions. The primers for real-time PCR analysis were designed with Primer-Blast based on the published cDNA sequence in the DNA bank or synthesized based on the primer sequences from publications (Table 5). All the primers were spanned for at least 2 exons. Then, all the primers were synthesized by Integrated DNA Technologies, Inc. (Coralville, Iowa). Real-time PCR (RT-PCR) was performed using SYBR Green Supermix (Bio-Rad) on a C.F.X. Connect Real-Time PCR Detection System (Bio-Rad). Briefly, 2 μL of cDNA was added to a total volume of 20 μL containing 10 μL SYBR Green mix and one μ. each of forwarding and reversed primers. Each of the samples was analyzed in duplicate for each gene expression. The following thermocycling amplification conditions were used: denaturation for 15 s at 95°C, annealing for 15 s at 56°C, extension for 30 s at 72°C, and repeating for 40 cycles. In addition, a melting curve program was conducted to confirm the specificity of each product. Real-time-PCR data were analyzed using the 2^−ΔΔCT^ method to calculate the relative fold change of the target gene with glyceraldehyde 3-phosphate dehydrogenase (GAPDH) as an internal control (Livak and Schmittgen, 2001).

#### Concentration of IgA

Frozen jejunal samples were ground using mortar and pestle in liquid nitrogen. An aliquot of pulverized jejunal tissue samples (0.12 ± 0.022 g) were placed into free-standing microcentrifuge tube (02-682-558, Thermo Fisher, Waltham MA) followed by addition of Tissue Protein Extraction Reagent (**T-PER**; sample weight × 15; 78510, Thermo Fisher, Waltham MA). Then, 0.1 ± 0.01 g of acid-washed glass beads (≤106 µm; G4649-100G, Sigma Aldrich, St. Louis, MO) were added and followed by homogenization with a bead mill for 2 cycles of 150-s at 3 m/s (15-340-163; Fisher Brand bead mill-24, Thermo Fisher, Waltham MA). Homogenized samples were then centrifuged at 10,000 × *g* for 15 min at 4°C. Supernatants of jejunal tissue homogenates and thawed plasma were used for assay for the concentration of IgA using commercial pig IgA ELISA kits according to the manufacturer instructions (Cedarlane Labs., Burlington, ON, Canada).

#### Total Bacterial and Short-Chain Fatty Acid (SCFA)

The total bacteria and SCFA that are lactic, acetic, propionic, iso-butyric, n-butyric, iso-valeric, and n-valeric in the ceca digesta were assayed at Alimetrics labs, Finland. The total bacteria were determined based on the detection and quantification of a fluorescent reporter signal that directly increased to amounts of PCR product in the reaction, as Christensen et al. (2022) described. The primer for the total bacteria was previously reported by Kettunen et al. (2017). The data was reported as a number of copies of 16S RNA per gram of sample. Short-chain fatty acids were derivatized to the respective phenyl esters using phenyl chloroformate reagent and analyzed by gas chromatography (Agilent Technologies, Santa Clara, CA) using pivalic acid (Sigma-Aldrich, St. Louis, MO) as an internal standard. The chromatography procedure used a glass column packed with 80/120 Carbopack B-DA/4% Carbowax stationary phase, helium as a carrier gas, and a flame ionization detector described previously by Apajalahti et al. (2019).

### Calculation and Statistical Analyses

Mortality-adjusted FCR was calculated using Equation 1. The breast yield was standardized for BW, and microbial data were log-transformed before statistical analyses. An average value was derived for the sampled two birds per pen. The apparent retention (**AR**) of components was calculated according to Kiarie et al. (2014).

(1) $$\left(\text{FCR}\right)=\;\frac{Feed\;Intake\;\left(FI\right)\;}{BWG\;of\;survivors\;and\;Mortalities}$$

Data were evaluated for outliers using the box and whisker method and subsequently subjected to a Mixed model of the GLIMMIX procedure of SAS (Enterprise Edition 9.4; SAS Inst. Inc., Cary, NC) with the pen and cage as the experimental units for the floor metabolism trials, respectively. The model had diet as the fixed effect for the floor pen and block (room) as the variable effect, whereas the cage trial had diet as the fixed effect. Independent t-tests and Tukey methodology separated LSMeans for mains and interactions effect, respectively. Coefficients for linear and quadratic effects of yeast response in YBC were generated using IML procedures of SAS, and all statistical significances were declared at *P* < 0.05.

## RESULTS

Table 2 shows the analyzed chemical composition of the experimental diets. Within phases, the analyzed crude protein and crude fat were comparable with formulated values. However, analyzed calcium and sodium showed some discrepancies with formulated values.

**Table 2:** Analyzed chemical composition of experimental diets, % as-fed basis.

Item	Starter (d 0–10)					Grower (d 11–24)					Finisher (d 25–42)				
Yeast^1^ inclusion, %	0	0.05	0.10	0.20	0.40	0	0.05	0.10	0.20	0.40	0	0.05	0.10	0.20	0.40
Dry matter	87.3	88.2	88.3	88.1	87.9	87.0	87.7	87.4	87.8	87.5	87.4	87.6	88.0	87.7	87.7
Crude protein	22.5	22.9	23.0	22.8	22.8	21.8	21.2	21.5	21.7	21.1	18.9	19.3	19.4	19.4	19.7
Crude fat	3.59	3.41	3.45	3.39	3.51	5.19	5.23	5.28	5.51	5.43	7.12	6.96	6.65	6.99	7.01
Starch	36.0	34.8	35.8	34.7	35.9	37.1	36.2	36.2	37.1	35.2	38.2	36.1	36.1	36.3	35.9
Calcium	0.76	0.77	0.81	0.78	0.75	0.65	0.72	0.66	0.68	0.68	0.68	0.67	0.64	0.63	0.68
Phosphorous	0.53	0.52	0.51	0.54	0.52	0.46	0.49	0.5	0.48	0.48	0.46	0.47	0.42	0.47	0.48
Potassium	1.09	1.01	1.00	1.01	1.01	0.86	0.90	0.92	0.9	0.87	0.79	0.87	0.81	0.87	0.89
Magnesium	0.19	0.19	0.19	0.20	0.19	0.18	0.19	0.19	0.19	0.19	0.18	0.19	0.18	0.19	0.19
Sodium	0.14	0.15	0.16	0.16	0.18	0.16	0.17	0.16	0.16	0.19	0.17	0.16	0.13	0.17	0.15

1 Enzymatically treated whole non-GMO *Saccharomyces cerevisiae* strain, 40% β-1.3/1.6 glucans and mannan oligosaccharides and 36% crude protein (Livalta TMCell HY40, AB AGRI, Peterborough, UK).

### Growth Performance, Mortality, and Breast Weight

Table 3 shows growth performance and breast weight data. The OBC were heavier (*P* < 0.01) than the YBC at hatch. In the starter phase (days 0–10), OBC had higher (*P* < 0.01) BWG and FI than YBC. Yeast supplementation increased BWG and FI quadratically (P ≤ 0.05) and improved FCR linearly (*P* = 0.03) in the starter phase. In the grower phase (days 11–24), OBC had higher (*P* < 0.01) BWG than YBC not fed yeast or fed a 0.40% yeast-based diet. The YBC fed 0.05, and 0.10% yeast had higher BWG than YBC birds fed 0 and 0.40% yeast-based diets but similar (*P* > 0.05) to OBC. The YBC-fed yeast ate less feed (*P* = 0.04) than OBC in the grower phase. Although FCR for OBC was similar (*P* > 0.05) to YBC in the grower phase, yeast quadratically improved (*P*=0.004) FCR in YBC. In the finisher phase (days 25-42), there was no difference in the final BW (day 42) (*P* > 0.05) between OBC and YBC fed ≥0.10% yeast. The final BW of OBC was 2,780 g/bird vs. 2,560, 2,700, 2,720, 2,810, 2,740 g/bird for YBC fed 0, 0.05, 0.10, 0.20, and 0.40% yeast, respectively. Yeast supplementation in YBC diets resulted in linear (*P* = 0.02) and quadratic (*P* = 0.005) increases on day 42 BW. There was no difference between OBC and YBC on BWG in the finisher phase. Yeast supplementation had linear (*P* = 0.004) and quadratic (*P* = 0.05) responses on finisher phase BWG.

**Table 3:** Primer sequences for RT-PCR analyses for jejunal gene expression.

Gene	Genbank accession number		Primer sequence (5′-3′)	Product size (bp)	References
GAPDH	NM_204305	F	ACTGTCAAGGCTGAGAACGG	100	
		R	CACCTGCATCTGCCCATTTG		Araujo et al. (2019)
IL-6	NM_204628.1	F	GAAATCCCTCCTCGCCAATCT	106	
		R	CCTCACGGTCTTCTCCATAAACG		Elnagar et al. (2021)
TNFα	NM_204267	F	CAGGACAGCCTATGCCAACAAG	114	
		R	GGTTACAGGAAGGGCAACTCATC		Bhatnagar et al. (2010)
EAAC-1	XM_424930.5	F	GATTGTTCTGAGCGCTGTCG	115	
		R	ACCAAAGGCATCTCCCAAG		Li et al. (2013)
B0AT-1	XM_419056	F	GCTCTACAGTGTTTGGAACCC	111	
		R	AAACTAGGCACACCAGCGAT		Wang et al. (2019)
SGLT-1	NM_001293240.1	F	ATGCTGCGGACATCTCTGTT	117	
		R	TCCGTCCAGCCAGAAAGAAT		Shimizu et al. (2018)
PepT-1	NM_204365	F	CTTTGGCTACCCCTTGAGCA	127	
		R	AAAGTTGTCATCCCACCGCA		Osmanyan et al. (2018)
ZO-1	XM_015278981.1	F	TATGCACAAGGAGGTCAGCC	97	
		R	TTGGCCGAAGCATTCCATCT		Wan et al. (2022)
OCLN	NM_205128.1	F	ACGGCAGCACCTACCTCAA	127	
		R	GGGCGAAGAAGCAGATGAG		Wan et al. (2022)
IL-10	AJ621614	F	CATGCTGCTGGGCCTGAA	94	
		R	CGTCTCCTTGATCTGCTTGATG		Elnagar et al. (2021)
SOD-1	NM 205064.1	F	TTGTCTGATGGAGATCATGGCTTC	98	
		R	TGCTTGCCTTCAGGATTAAAGTGAG		Araujo et al. (2019)

Abbreviations: F, Forward primer; R, Reverse primer.

B0AT1, Sodium-dependent neutral amino acid transporter; EAAC1, Excitatory amino-acid carrier 1; GAPDH, Glyceraldehyde 3-phosphate dehydrogenase; IL-6, Interleukin-6; OCLN, Occuludin; PEPT1, Peptide transporter 1; SGLT1, Sodium-glucose transporter 1; SOD1, Superoxide dismutase 1; TNF-α, Tumor necrosis factor-α; ZO 1, Zonula occludens-1.

The YBC fed 0.20 and 0.40% yeast exhibited lower feed intake (*P* = 0.02) and better FCR (*P* = 0.003) than OBC. Supplemental yeast linearly decreased FCR (*P* = 0.006). Overall (days 0–42), the BWG of older breeder birds was higher (*P* = 0.024) than for younger birds fed 0 or 0.05% yeast but similar (*P* > 0.05) to birds fed yeast. Supplemental yeast increased overall BWG linearly (*P* = 0.021) and quadratically (*P* = 0.005). OBC and YBC fed 0.05, and 0.10% yeast consumed an equivalent (*P* > 0.05) amount of feed. However, OBC ate more (*P* = 0.008) than YBC fed 0 or ≥0.20% yeast. The OBC had similar FCR (*P* > 0.05) to YBC fed 0 to 0.10% yeast but higher (*P* = 0.003) than for YBC fed ≥0.20% yeast. Among the YBC, yeast had linear (*P*=0.03) and quadratic (*P* = 0.01) improvement in overall FCR. In this context, supplementing 0.05, 0.10, 0.20, and 0.40% yeast improved overall FCR by 7, 8, 17, and 11 points, respectively. Although overall mortality (days 0–42) did not differ (*P* = 0.09) between birds of OBC and YBC, OBC showed numerically lower mortality than YBC. Supplementation of yeast linearly (*P*=0.02) reduced mortality. The OBC had heavier (*P* = 0.02) breast weight than YBC; however, yeast supplementation increased breast weight in YBC linearly (*P* = 0.02) and quadratically (*P* = 0.04).

### Organ Weight, Jejunal Histomorphology, Gene Expression, and Concentration of IgA

Data for the organ weight, jejunal histomorphology, gene expression, and the concentration of IgA and primer sequences are shown in Tables 4 and 5, respectively. Treatments had no effects (*P* > 0.05) on day ten liver weight. The OBC and YBC fed 0, 0.10, and 0.40% yeast had similar but lighter (*P* = 0.04) spleen than birds fed 0.05 and 0.20% yeast. Older breeder chicks had heavier (*P* = 0.004) bursa than YBC fed 0, 0.1, and 0.40% yeast. Supplemental yeast had quadratic effects on the spleen (*P* = 0.06) and bursa (*P* = 0.05). The bursa was 12.4, 1.7, 17.9 10.6% heavier for 0.05, 0.1, 0.2, and 0.4 yeast, respectively. Birds fed 0.4% yeast had taller (*P* < 0.001) jejunal villi than birds of other treatment groups. There were no treatment effects on jejunal crypt depth. Although there was no difference between OBC and YBC on VCR, yeast increased VCR linearly (*P* = 0.002) and quadratically (*P* = 0.006) in YBC. Younger breeder chicks exhibited higher (*P* < 0.001) jejunal expression of sodium-dependent neutral amino acid transporter (B0AT1) than OBC. Among the YBC, yeast linearly increased (*P* < 0.001) jejunal expression of B0AT1. Treatments had no (*P* > 0.05) effects on the expression of other nutrient transporters assessed, which are excitatory amino-acid carrier 1, peptide transporter 1, and sodium-glucose transporter 1 (EAAC1, PepT1, SGLT1). Treatments had no effects on the expression of interleukin-6 (IL6) (*P* = 0.14) and interleukin-10 (IL10) (*P* = 0.21). However, yeast inclusion quadratically reduced IL6 expression in YBC (*P* = 0.030). Although OBC and YBC showed similar jejunal expression of tumor necrosis factor-α (**TNF-α**), yeast quadratically (*P* = 0.01) reduced TNF-α. There were no (*P* > 0.05) treatment effects on zonula occludens-1 (**ZO1**) expression. Although OBC and YBC showed similar (*P* > 0.05) jejunal expression of occuludin (**OCLN**), yeast increased its expression quadratically (*P* = 0.002) in YBC.

**Table 4:** Effects of broiler breeder age and dietary yeast fed to young breeder chicks on growth performance through to 42 d of age.^1^

	Older breeder chicks	Younger breeder chicks (YBC)					SEM	*P*-value	Yeast response in YBC	
Yeast^2^ inclusion, %:	0	0	0.05	0.10	0.20	0.40			Linear	Quadratic
Starter^3^ phase, days 0-10										
Initial BW, g/bird	43.2^a^	39.5^b^	39.2^b^	39.2^b^	39.2^b^	39.2^b^	0.20	<0.01	-	-
Day 10 BW, g/bird	272.2^a^	234.5^c^	244.6^b^	247.8^b^	246.1^b^	243.2^b^	3.02	<0.01	0.288	0.014
Body weight gain, g/bird	229.0^a^	195.0^c^	205.4^b^	208.6^b^	206.9^b^	204.0^b^	2.92	<0.01	0.261	0.014
Feed intake, g/bird	246.8^a^	219.4^c^	227.6^b^	230.1^b^	225.5^bc^	223.3^bc^	2.80	<0.01	0.883	0.051
FCR	1.091^c^	1.159^a^	1.144^ab^	1.127^abc^	1.120^bc^	1.096^c^	0.013	0.003	0.030	0.531
Grower phase, days 11-24										
Day 24 BW, g/bird	1,115^a^	994.7^c^	1,077^ab^	1,070^ab^	1,037^bc^	1004^c^	19.8	0.001	0.227	0.024
Body weight gain, g/bird	842.5^a^	760.2^b^	832.0^a^	822.2^a^	790.7^ab^	760.5^b^	19.0	0.009	0.159	0.052
Feed intake, g/bird	1,129^a^	1,052^ab^	1,043^b^	1030^b^	987.4^b^	1,046^b^	28.6	0.041	0.773	0.122
FCR	1.343^ab^	1.387^a^	1.261^b^	1.268^b^	1.263^b^	1.383^b^	0.034	0.017	0.338	0.004
Finisher phase, day 25-42										
Day 42 BW, g/bird	2,780^a^	2,565^b^	2,702^ab^	2,722^a^	2,807^a^	2,741^a^	49.0	0.023	0.022	0.005
Body weight gain, g/bird	1,665^abc^	1,571^c^	1,626^bc^	1,652^abc^	1,770^a^	1,737^ab^	43.9	0.032	0.004	0.054
Feed intake, g/bird	2,771^a^	2,571^ab^	2,592^ab^	2,597^ab^	2499^b^	2,543^b^	78.6	0.024	0.578	0.738
FCR	1.673^a^	1.641^a^	1.605^ab^	1.580^ab^	1.436^c^	1.478^bc^	0.044	0.003	0.006	0.080
verall, days 0-42										
Body weight gain, g/bird	2,737^a^	2,526^b^	2,663^ab^	2,683^a^	2,767^a^	2,701^a^	49.0	0.024	0.021	0.005
Feed intake, g/bird	4,147^a^	3,843^b^	3,862^ab^	3,857^ab^	3,712^b^	3,812^b^	102.4	0.008	0.607	0.516
FCR	1.516^a^	1.521^a^	1.453^ab^	1.440^ab^	1.353^c^	1.416^bc^	0.030	0.003	0.027	0.008
Mortality, %	1.98	3.71	3.98	3.41	3.70	1.137	0.804	0.091	0.017	0.264
Breast weight, day 42										
g/kg BW	229^a^	212^b^	226^a^	228^a^	231^a^	231^a^	4.326	0.018	0.019	0.037

1 Older breeder, 47 wk of age; younger breeder, 30 wk of age.

2 Enzymatically treated whole non-GMO *Saccharomyces cerevisiae* strain, 40% β-1.3/1.6 glucans and mannan oligosaccharides and 36% crude protein (Livalta TMCell HY40, AB AGRI, Peterborough, UK).

3 LSmeans with different superscript letters^a,b,c^ within a row differs, *P* < 0.05.

**Table 5:** Effects of broiler breeder age and dietary yeast fed to young breeder chicks on organ weights, jejunal histomorphology, expression of select genes, and concentration of IgA in plasma and jejunal tissues at ten days of age.^1^

	Older breeder chicks	Younger breeder chicks (YBC)					SEM	*P*-value	Yeast response in YBC	
Yeast^2^ inclusion (%):	0	0	0.05	0.1	0.2	0.4			Linear	Quadratic
Organ weight, g/kg BW^3^										
Liver	37.1	38.1	37.7	38.4	38.6	38.7	1.08	0.908	0.581	0.918
Spleen	0.723^b^	0.757^b^	0.938^a^	0.826^ab^	0.941^a^	0.880^ab^	0.06	0.038	0.353	0.062
Bursa	1.913^a^	1.469^c^	1.677^abc^	1.495^c^	1.789^ab^	1.644^bc^	0.09	0.004	0.205	0.052
Histomorphology										
Villi height (VH), µm	1,070.0^bc^	952.4^c^	1,049.3^bc^	1,147.2^ab^	1,169.6^ab^	1,226.9^a^	32.2	<0.0001	<0.0001	0.006
Crypt depth (CD), µm	153.5	173.9	146.7	147.8	139.8	149.3	9.97	0.257	0.237	0.052
VH:CD	7.12^ab^	5.67^b^	7.39^ab^	8.05^a^	8.40^a^	8.40^a^	0.46	0.001	0.002	0.006
Transporters										
B0AT1	0.63^b^	1.34^a^	1.35^a^	1.60^a^	1.82^a^	1.77^a^	0.16	<0.0001	0.034	0.203
EAAC1	2.20	2.39	2.28	2.77	2.53	2.74	0.22	0.3671	0.239	0.733
PepT1	3.05	3.17	2.53	3.05	2.90	2.78	0.20	0.2754	0.573	0.850
SGLT1	0.44	0.75	0.65	0.59	0.64	0.56	0.12	0.5792	0.413	0.719
Cytokines										
Il6	10.31	11.48	9.83	10.20	9.97	10.36	0.44	0.139	0.339	0.030
IL10	12.75	13.05	13.36	13.71	14.05	13.45	0.38	0.212	0.577	0.156
TNF-α	1.54^ab^	2.20^a^	0.97^b^	1.45^ab^	1.56^ab^	1.94^a^	0.19	0.001	0.253	0.006
Tight junction proteins										
OCLN	5.80^ab^	4.98^b^	5.56^ab^	6.30^a^	5.94^a^	5.54^ab^	0.21	0.0025	0.404	0.002
ZO1	5.38	5.24	5.38	5.75	5.52	5.57	0.18	0.4207	0.336	0.270
Oxidative stress										
SOD	0.49^c^	0.72^bc^	1.05^ab^	1.22^a^	1.30^a^	1.39^a^	0.10	<0.0001	<0.0001	0.011
Plasma IgA (mg/mL)	241.3^b^	243.0^b^	325.2^ab^	342.2^ab^	384.1^a^	387.9^a^	28.0	0.001	0.001	0.020
Jejunal IgA (ng/mL)	2,680^ab^	2,039^b^	1,959^b^	2,768^ab^	2,800^ab^	3474^a^	204	0.001	0.001	0.043

1 Older breeder, 47 wk of age; younger breeder, 30 wk of age.

2 Enzymatically treated whole non-GMO *Saccharomyces cerevisiae* strain, 40% β-1.3/1.6 glucans and mannan oligosaccharides and 36% crude protein (Livalta TMCell HY40, AB AGRI, Peterborough, UK). B0AT1, Sodium-dependent neutral amino acid transporter; EAAC1, Excitatory amino-acid carrier 1; IL-6, Interleukin-6; OCLN, Occuludin; PEPT1, Peptide transporter 1; SGLT1, Sodium-glucose transporter 1; SOD1, Superoxide dismutase 1; TNF-α, Tumor necrosis factor-α; ZO 1, Zonula occludens-1.

3 LSmeans with different superscript letters^a,b,c^ within a row differs, *P* < 0.05.

Regarding the marker gene expression for oxidative stress, OBC and YBC not fed yeast exhibited similar (*P* > 0.05) expression of superoxide dismutase 1 (**SOD1**). Yeast supplementation, however, increased the expression of SOD1 in linear (*P* < 0.01) and quadratic (*P* = 0.01) fashions. Neither plasma IgA nor jejunal IgA concentrations differed between OBC and YBC-fed yeast-free diets. However, the plasma IgA concentration of birds fed 0.2 and 0.4% yeast was higher (*P* = 0.001) than OBC and yeast-free YBC. In addition, yeast had a linear (*P* = 0.001) and quadratic (*P* ≤ 0.04) increase in the concentration of IgA in plasma and jejunal tissue samples.

### Ceca Microbial Activity and Apparent Metabolizable Energy

Treatments had no (*P* > 0.05) effects on total bacteria and concentration of SCFA in the ceca digesta of 24-day-old broiler chickens (Table 6). However, there was a tendency (*P* = 0.07) for OBC to show a lower concentration of ceca digesta total bacteria than YBC. Similarly, treatment had no (*P* > 0.05) effects on litter moisture on days 35 and 42 (Table 6). In addition, there was no (*P* > 0.05) effect of breeder age on apparent retention of dry matter, crude protein, gross energy, and apparent metabolizable energy (**AME**) (Table 7). However, yeast supplementation in YBC led to a linear and quadratic (*P* < 0.01) increase in apparent retention of dry matter, crude protein, and gross energy.

**Table 6:** Effects of broiler breeder age and dietary yeast fed to young breeder chicks on the concentration of total bacteria and short-chain fatty acids (SCFA) in ceca digesta at 24 d of age and litter moisture content at days 35 and 42 of age.^1^

	Older breeder chicks	Younger breeder chicks (YBC)					SEM	*P*-value	Yeast response in YBC	
Yeast^2^ inclusion (%):	0	0	0.05	0.10	0.20	0.40			Linear	Quadratic
Total, bacteria log_10_, 16 sRNA copies/g	12.1	12.4	12.4	12.4	12.3	12.4	0.07	0.069	0.649	0.148
SCFA, mmol/kg										
Acetic	57.6	58.3	65.7	52.6	60.0	57.0	5.90	0.743	0.723	0.951
Propionic	3.40	3.63	3.66	4.14	3.42	3.32	0.43	0.787	0.416	0.737
Butyric	11.5	13.5	13.6	13.4	12.1	11.5	1.60	0.850	0.293	0.947
Valeric	0.91	1.01	0.93	0.97	0.85	0.89	0.09	0.803	0.324	0.491
Lactic	0.74	0.85	0.80	0.91	0.94	0.93	0.17	0.951	0.620	0.796
BCFA	1.16	1.11	0.94	1.14	0.92	1.12	0.15	0.754	0.848	0.446
VFA	74.6	77.6	84.8	72.3	77.3	73.9	7.43	0.869	0.573	0.947
Total SCFA	75.3	78.4	85.6	73.2	78.2	74.8	7.43	0.872	0.581	0.952
SCFA: BCFA	72.9	79.8	95.3	79.0	102.1	79.0	14.3	0.695	0.899	0.356
Litter moisture, %										
Day 35	27.5	26.1	26.4	24.4	23.9	26.4	1.32	0.413	0.376	0.539
Day 42	29.1	29.7	29.6	27.1	26.3	27.7	1.34	0.368	0.215	0.096

1 Older breeder, 47 wk of age; younger breeder, 30 wk of age.

2 Enzymatically treated whole non-GMO Saccharomyces cerevisiae strain, 40% β-1.3/1.6 glucans and mannan oligosaccharides and 36% crude protein (Livalta TMCell HY40, AB AGRI, Peterborough, UK). LSmeans with different superscript letters differ within a row, *P*<0.05. Netterville, Dowth, Co Meath, Ireland A92 ER22.

**Table 7:** Effects of broiler breeder age and dietary yeast fed to young breeder chicks on apparent retention of components at 21 d of age.^1^

Item	Yeast^2^ inclusion, %	Apparent retention, %		
		Dry matter	Crude protein	Gross energy
Older breeder chicks^3^	0.0	70.8^b^	73.1^bc^	77.4^b^
Younger breeder chicks				
	0.0	67.8^b^	68.5^c^	74.7^b^
	0.05	71.7^b^	74.0^ab^	77.9^b^
	0.1	76.9^a^	78.4^a^	82.0^a^
	0.2	76.8^a^	78.2^a^	82.0^a^
	0.4	77.8^a^	79.1^a^	82.7^a^
SEM	-	1.58	1.77	1.26
*P*-value	*-*	<0.01	<0.01	<0.01
Yeast response in YBC				
Linear	-	<0.01	0.001	<0.01
Quadratic	-	0.005	0.009	0.003

1 Older breeder, 47 wk of age; younger breeder, 30 wk of age.

2 Enzymatically treated whole non-GMO Saccharomyces cerevisiae strain, 40% β-1.3/1.6 glucans and mannan oligosaccharides and 36% crude protein (Livalta TMCell HY40, AB AGRI, Peterborough, UK).

3 LSmeans with different superscript letters^a, b, c^ differ within a column differs, *P* < 0.05.

## Discussion

The goal of broiler breeder farm is to produce as many quality eggs as possible to maximize chicks per hen placed. On the other hand, broiler chicken farmer, desires fast-growing and uniform chicks that are resilient to growing conditions and achieve yield targets with minimal feed costs. In this context, chick robustness and quality are critical attributes in the broiler breeder-hatchery-grow out value chain. Chick quality is determined by many factors, such as breeder genetic line, age, nutrition, and housing conditions, as well as the hatchery processes (Ulmer-Franco et al., 2010; Duman and Şekeroğlu, 2017). However, broiler breeder age has a huge influence, and comparative data from chicks of younger and older broiler breeders shows differences in hatch weight, growth, intestinal development, resistance to enteric diseases and immunocompetence (Ulmer-Franco et al., 2010). As such the hatch body weight, early phase growth and feed intake of OBC were significantly higher than for YBC in the current study. These observations corroborated earlier studies that reported that older broiler breeders hatched bigger chicks (PawŁowska and Sosnówka-Czajka, 2019) that grew faster compared to chicks from younger breeders (Nangsuay et al., 2013). Larger eggs have proportionally heavier yolk. As the primary source of embryo nourishment, the yolk contributes to chick size at hatch. Overall, younger breeder hatchling reflects a smaller proportion of nutrients and factors in the egg.

The current study assessed whether feeding YBC enzymatically treated yeast could bridge the growth performance gap with OBC. Supplemental yeast stimulated feed intake, aligning with other studies that reported yeast and yeast derivatives increased feed consumption in broiler chickens (Zhang et al., 2005; Morales-López et al., 2010). Studies have found that yeast and yeast derivatives can enhance and growth and FCR in broiler chickens (Ghosh et al., 2012; Zhang et al., 2012). In our previous study, the yeast tested in the current study improved growth performance in broiler chickens and piglets (Christensen et al., 2022; Kiarie et al., 2022). Perhaps stimulation of feed intake and nutrient utilization in YBC partly explains why YBC caught up with the OBC in later phases. In addition, an increased body weight gain translated to heavier breasts in yeast fed YBC. Other studies found that supplementation of yeast derivatives resulted in heavier breasts (Aristides et al., 2018; Arif et al., 2020; Wang et al., 2022). It should be noted that, there are many yeasts associated feed ingredients and feed additives that are produced, marketed, and applied in animal agriculture around the world (Shurson, 2018); an aspect that makes comparison of studies very difficult. Moreover, there are numerous studies that reported no or small effect of yeast supplementation on growth performance in broiler chickens. For example, a meta-analysis of 29 experiments testing yeast cell wall fed broilers versus control showed a modest (<2%) impact on growth performance (Hooge, 2004). Interestingly, the same meta-analyses indicated more than 20% reduction in mortality in birds fed yeast cell walls relative to the control. Similarly, yeast linearly reduced overall mortality in the current study.

Liver weight as an indicator of metabolic capabilities revealed no difference between OBC and YBC. Suggesting, growth differences in starter phase were not due to nutrient metabolism. There is data to suggest that yeast supplementation not only affects immunity and growth in animals but also metabolic responses (Sanchez et al., 2021). However, this was not evident in the current study in YBC fed yeast. However, it is noteworthy that YBC fed yeast showed higher nutrients and energy retention. The size of lymphoid organ reflects animal ability to provide lymphoid cells during an immune response and immunosuppressed birds have smaller lymphoid organs (Pope, 1991). Spleen primary functions are to filter blood and agents as well as provide environment for the proliferation and maturation of cells involved in the adaptive immune response. The Bursa of Fabricius is considered the primary lymphoid organ in poultry and is critical in differentiating B-lymphocytes. Comparatively, OBC had heavier bursa than YBC birds than birds fed 0, 0.1, and 0.4% yeast. However, among YBC, yeast had tendency for quadratic responses on these lymphoid organs. Various studies reported that yeast derivatives modulated lymphoid organs in broiler chickens (Lu et al., 2019; Sjofjan et al., 2021). These effects have been linked to stimulation of the gut-associated immune system (Kiarie et al., 2019). However, the response of supplemental yeast on enlargement of lymphoid organs is variable. For example, feeding various forms and dosages of yeast products increased weight of lymphoid organs (Guo et al., 2003; Zhang et al., 2008. Morales-Lopez et al. 2010; Zhang et al., 2012) but no effects in other studies (Rathgeber et al., 2008).

Functional gastrointestinal tract is important in sustaining growth performance (Reisinger et al., 2012; Shao et al., 2013; Alizadeh et al., 2016). The OBC had similar jejunal histomorphology indices (VH and VCR) and apparent retention of DM and GE to nonyeast YBC. Suggesting superior growth in OBC was not related to differences in digestive capacity. Zhang et al. (2005) found that yeast bioactives increased intestinal villi height and crypt depth. Instructively, YBC fed the highest dose of yeast had taller villi than OBC and birds fed yeast retained more energy than OBC. The YBC had higher expression of B0AT1 than OBC and yeast linearly increased expression among YBC. This corresponded with higher (linear) retention of crude protein and gross energy in birds fed yeast. These observations suggested yeast supplementation enhanced nutrients utilization to catch up with OBC in terms of growth. Tumor necrosis factor (**TNFα**) expression was quadratically reduced by yeast perhaps linked to yeast components activation of phagocytes macrophages and monocytes (Silva et al., 2009; Eom et al., 2021). Yeast increased occludin expression an indication of role in gut permeability (Shen and Turner, 2006; von Buchholz et al., 2021). Physiological oxidative stress in broiler chickens is indicated by superoxide dismutase (SOD1). Although birds in the present study were not subjected to stressful conditions, SOD1 expression linearly increased with supplemental yeast. We theorize that yeast bioactives increased metabolic activities at the gut level implication that requires further investigation.

Enteric pathogens loads are significant factor in the broiler chickens production (Chapman et al., 2016; Kim et al., 2017). A healthy gut has been associated with low pathogen loads primarily of commercial concern, such as *Eimeria, Coccidiosis,* and *Clostridium* (Luo et al., 2013), and consequently low flock disease burden. Other than vaccinations, immunity indices can be modulated through feeding bioactives such as yeast and yeast derivatives (Szczypka et al., 2021; Vailati-Riboni et al., 2021). We observed that feeding yeast to YBC increased jejunal and plasma IgA, an immune protein associated with health resilience, especially concerning the humoral immune system of the intestinal environment. Yeast bioactives have been reported to stimulate production of intestinal IgA (Corthésy, 2013; Huff et al., 2013; Gutzeit et al., 2014; Alizadeh et al., 2016). Although yeast and yeast derivatives have been reported to increase short-chain fatty acid cecal fermentation (Bortoluzzi et al., 2018). Yeast had no effect on SCFA in the current study, and in our previous studies that tested the same product in broiler chickens (Kiarie et al., 2022) and (Christensen et al. 2022). In general, SCFA are metabolites generated by bacterial fermentation of undigested nutrients flowing in the ceca. In alignment with AR of DM and total bacteria count, it is plausible there was limited availability of fermentable substrates to trigger differences in SCFA output.

With respect to yeast application, accurate dosing is essential for achieving desired benefits and preventing excessive feeding of biologically active components (Kiarie et al., 2019). In the current study, yeast supplementation elicited linear and non-linear responses suggesting correct dosing and elucidation of host response at cellular level requires further investigations. Moreover, although yeast feed additives are widely used in the feed industry, there is paucity of methodologies for active metabolite quantification in the feed which is critical for accurate dosing. A higher dose of up to 0.5% of yeast supplement was reportedly feasible (Perricone et al., 2022). However, the unintended negative effects of feeding higher levels of yeast and yeast bioactives are possible. For example, the yeast cell wall mannans that have been found to be antinutritional (Blibech et al., 2019; Chen et al., 2021). Over production of immune proteins and stimulation of lymphoid organs require nutrients that would have otherwise supported growth. Moreover, whole yeast is rich in nucleic acids that can result in elevated plasma urea N that is energetically costly to excrete (de Oliveira and Burini, 2012; Kiarie et al., 2020).

Typical to scientific investigations, the current study had some limitations worthy taking into considerations. Although the 2 groups of hatching eggs were procured from the same commercial farm and were of the same genetic line, this information was to the extent guaranteed by the supplier. The researchers had no access to specific information on how the parent flocks were fed and managed prior to collection of eggs. Nonetheless, the information contributes to emerging concepts of integrating broiler breeder physiology in the solutions for contemporary issues in broiler chickens production. The profitability and sustainability of broiler meat production chain largely relies on healthy and quality chicks. The current study showed that feeding enzymatically treated yeast to broiler chicks hatched from young breeders increased growth performance. Although not all health indices were influenced by yeast supplement, enhancement of indices of intestinal function could be attributed to improved growth and breast weight in YBC. Therefore, enzymatically treated yeast should be considered helpful in improving the growth performance of broiler chicks from younger breeder flock.

## Appendix Supplementary materials

Image, application 1

## Footnotes

Supplementary material associated with this article can be found in the online version at doi:10.1016/j.psj.2022.102175.
