# Supplementary material for: Enzymatically treated yeast bolstered growth performance of broiler chicks from young broiler breeders linked to improved indices of intestinal function, integrity, and immunity
Source: Poult Sci. 2022 Sep 13;101(12):102175. doi: 10.1016/j.psj.2022.102175 (PMC9573925; doi:10.1016/j.psj.2022.102175)
Supplement: Supplementary file 1 [file mmc1.docx]

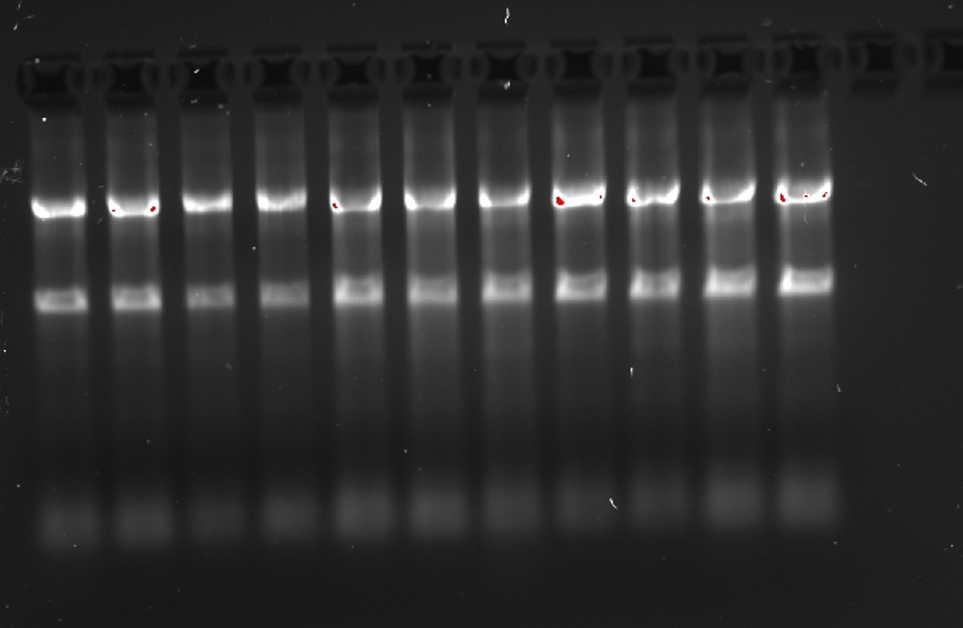


**Supplementary Figure 1**. Agarose gel visualizing RNA from a select number of jejunal tissue samples.
